# A circular RNA derived from GLIS3 accelerates the proliferation of glioblastoma cells through competitively binding with miR-449c-5p to upregulate CAPG and GLIS3

**DOI:** 10.1186/s12868-022-00736-6

**Published:** 2022-09-16

**Authors:** Qingjiu Zhou, Mahati Shaya, Yalikun Kugeluke, Qiang Fu, Shaoshan Li, Yisireyili Dilimulati

**Affiliations:** 1 Department of Neurosurgery, The First Affiliated Hospital of Xinjiang Medical University, No.137 South Liyushan Road, Ürümqi, 830054 Xinjiang China; 2 Department of Oncology, The First Affiliated Hospital of Xinjiang Medical University, Ürümqi, 830054 Xinjiang China; 3 Department of Neurosurgery, Yili Friendship Hospital, Yili, 835000 Xinjiang China

**Keywords:** circGLIS3, miR-449c-5p, CAPG, GLIS3, Glioblastoma

## Abstract

**Background:**

Glioblastoma (GBM) is an aggressive and malignant brain tumor with extremely poor prognosis. Despite advances in treatment, the pathogenesis of GBM remains elusive. Mounting studies have revealed the critical role of circular RNAs (circRNAs) in the development and progression of human cancers including GBM, but the comprehension of their functions is still insufficient. In this study, we investigated the expression profile of a circRNA derived from GLIS family zinc finger 3 (GLIS3) in GBM and normal astrocytes. CircGLIS3 expression was detected through quantitative real-time polymerase chain reaction (qRT-PCR) analysis. Functional experiments were performed to analyze the influence of circGLIS3 on GBM cell proliferation and apoptosis. In addition, mechanism assays were to uncover the potential regulatory mechanism of circGLIS3.

**Results:**

CircGLIS3 was up-regulated in GBM cells and knockdown of circGLIS3 significantly hampered proliferation and promoted apoptosis of GBM cells. Furthermore, circGLIS3 positively regulated CAPG and GLIS3 by sponging miR-449c-5p to affect GBM cell proliferation and apoptosis.

**Conclusions:**

In summary, our study identified that circGLIS3 could promote proliferation and inhibit apoptosis of GBM cells via targeting miR-449c-5p/GLIS3/CAPG axis in vitro. This study could offer a novel molecular perspective for further investigation into mechanisms essential to GBM progression.

**Supplementary Information:**

The online version contains supplementary material available at 10.1186/s12868-022-00736-6.

## Introduction

As one of the most fatal neurologic cancers around the world, glioblastoma (GBM) has always been a difficult problem for the medical community [1]. Previous research has indicated that patients with GBM have a median survival of roughly 15 months, and the 5-year survival rate is about 5% [2]. Although advancement has been achieved in therapy of GBM, mortality caused by GBM is still significantly high [3]. Based on the current evidence, some molecular pathways are related to GBM pathogenesis, including small non-coding molecules like miR-21 [4], NF-kappaB pathway [5] and growth factor receptors [6]. However, rapid invasion of the brain parenchyma, resistance to conventional therapy and drug resistance severely lessen the effectiveness of treatment regimens [7]. Therefore, a better grasp of GBM pathogenesis is crucial for developing available therapeutic markers and targets [8–10].

In recent years, circular RNA (circRNA) becomes a rising focus in the field of biological research [11–14]. Compared with traditional linear RNAs, single-stranded, covalently closed circRNAs have a longer half-life and are more resistant to RNase R treatment, providing them the potential to serve as diagnostic biomarkers and therapeutic targets [15]. As circRNA molecules have a large number of miRNA binding sites, circRNAs can serve as miRNA “sponges”, which could competitively binding to miRNAs and lead to decreased functional miRNA molecules and up-regulation of target genes [16]. Via RNA sequencing, more than 80,000 circRNAs have been identified [13]. The association between circRNAs differentially expressed in GBM tissues, and the GBM progression has been unveiled [17]. For instance, circSCAF11 promotes GBM progression by sponging miR-421 and modulating SP1 and VEGFA [18]. Additionally, circDENND2A/miR-625-5p signaling can accelerate GBM development [19]. The above results demonstrated the importance of circRNAs in the development of GBM. However, to implement more precise therapy of GBM, more circRNAs need to be investigated [20]. Specifically, circGLIS3 (circ_0005890) has a length of 94,738 bp and the gene that codifies for circGLIS3 is located at chr9: 4,191,785–4,286,523. Previous study has identified that circGLIS3 was an oncogene in GBM [21]. Hinted by previous study unveiling that circGLIS3 could promote non-small cell lung cancer via regulating miR-644a/PTBP1 axis [22], we also intended to identify the functional role of circGLIS3 in GBM and figure out the underlying regulatory mechanism.

The interaction between circRNAs and their host genes has been found to play a crucial role in GBM. Specifically, circ-EPB41L5 could sponge miR-19a to regulate its host gene EPB41L5 to suppress GBM tumorigenesis [20]. GLIS3, the host gene of circGLIS3, is a member of GLI-similar zinc finger protein family and encodes a nuclear protein with five cysteine 2/histidine 2 (Cys2/His2) zinc finger domains [23]. Via binding with GLIS3-binding sites of target genes, GLIS3 can control gene transcription [24]. GLIS3 is overexpressed in several human organs, such as kidney, thyroid, and pancreas [25]. In this study, the potential involvement of GLIS3 in the regulatory mechanism of circGLIS3 in GBM cells was also explored. Capping actin protein, gelsolin-like (CAPG) encodes a member of the gelsolin/villin family of actin-regulatory proteins. Based on recent researches, CAPG was found to be overexpressed in GBM and could be a potential biomarker for GBM diagnosis [26]. Therefore, we also intended to investigate the potential correlation between circGLIS3 and CAPG in GBM cells.

In the current study, we aimed to verify the role of circGLIS3 in GBM cells and the relevant regulatory mechanism. Our findings will reveal a novel regulatory mechanism of circGLIS3 in GBM cells, contributing to better understanding of GBM.

## Results

### CircGLIS3 is up-regulated in GBM cells

According to previous study on circular RNA profiling in gliomas [27], circGLIS3 has been identified to be up-regulated in GBM tissues relative to deceased normal controls. CircBase database (http://www.circbase.org/) showed that there were 16 isoforms of circGLIS3. We utilized quantitative real-time polymerase chain reaction (qRT-PCR) analysis to detect the expression levels of the 16 isoforms in GBM cells and normal astrocytes (NHAs). The result of qRT-PCR clearly demonstrated that circ_0005890 was most aberrantly up-regulated in GBM cells (Additional file 1: Fig. S1A). Then, we designed experiments to validate whether circGLIS3 was a real circRNA. As shown in Additional file 1: Fig. S1B, circ_0005890 (circGLIS3) was derived from GLIS3 through back-spliced junction of intron 2, and the result of Sanger sequencing confirmed the head-to-tail splicing in the RT-PCR product of circGLIS3. After designing cDNA and gDNA primers for circGLIS3 and GAPDH on Primer-BLAST (https://www.ncbi.nlm.nih.gov/tools/primer-blast/), we conducted PCR-AGE assay. As was depicted in Additional file 1: Fig. S1C, circular transcript of circGLIS3 in cDNA but not gDNA was detected by divergent primers. Meanwhile, convergent primers magnified linear transcript from both cDNA and gDNA. Furthermore, linear GLIS3 mRNA but not circGLIS3 was digested by RNase R treatment (Additional file 1: Fig. S1D). To summarize, we found that as a real circRNA, circGLIS3 was up-regulated in GBM cell lines relative to the noncancerous cell line.

### Knockdown of circGLIS3 inhibits the proliferation and promotes the apoptosis of GBM cells

**Fig. 1:**
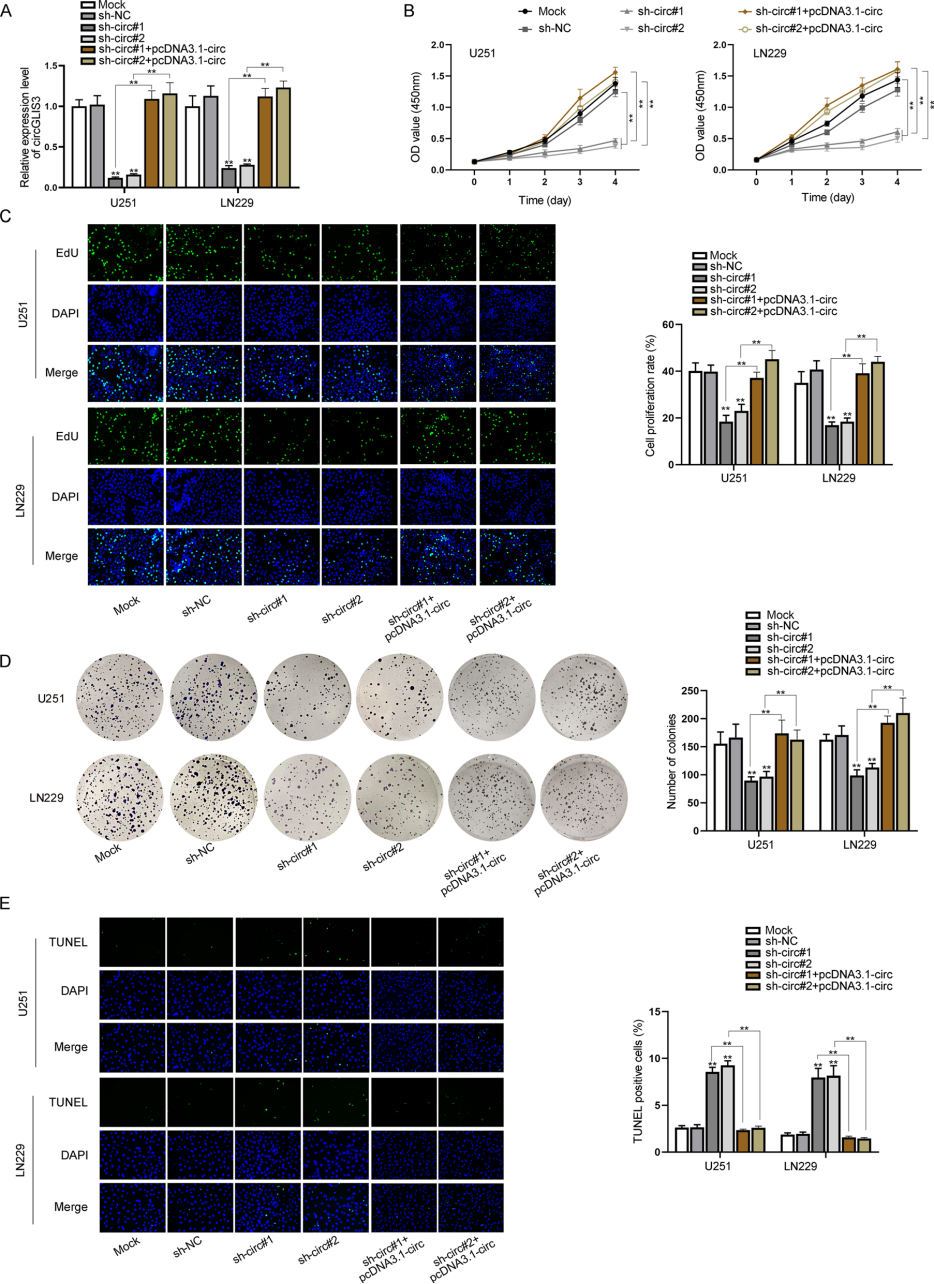
GBM cell proliferation and apoptosis are regulated by interfering circGLIS3. **A** qRT-PCR detected the expression of circGLIS3 in cells transfected with sh-circ or co-transfected with sh-circ + pcDNA3.1-circ. **B**, **C** Proliferation of cells transfected with sh-circGLIS3 or co-transfected with sh-circ + pcDNA3.1-circ was analyzed by CCK-8 and EdU assays. **D** Colony formation assay was implemented to test cell proliferation of U251 and LN229 transfected with sh-circGLIS3 or co-transfected with sh-circ + pcDNA3.1-circ. **E** The apoptotic rate of U251 and LN229 cells after transfection of sh-circGLIS3 or co-transfection with sh-circ + pcDNA3.1-circ was measured through TUNEL assay. One-way ANOVA was applied for statistical analysis. **P < 0.01

To begin with, we obtained sh-circ#1/2 for knocking down circGLIS3 in U251 and LN229 cells. Additionally, to detect whether there was off-target effect of sh-circ#1/2, pcDNA3.1-circ was also procured to overexpress circGLIS3 in sh-circ#1/2 transfected cells. The overexpression efficiency was first detected through qRT-PCR (Additional file 1: Fig. S2A). As shown in Fig. 1A, compared with the control group, expression of circGLIS3 in U251 and LN229 cells transfected with sh-circ#1 and sh-circ#2 was much lower, and pcDNA3.1-circ co-transfection could completely rescue the decreased expression of circGLIS3. Then, function experiments were conducted. From the result of cell counting kit-8 (CCK-8) assay, decline in cell viability was caused by transfection of sh-circ#1 and sh-circ#2, and pcDNA3.1-circ co-transfection could offset the effect (Fig. 1B). Moreover, 5-ethynyl-2ʹ-deoxyuridine (EdU) assay showed that upon sh-circ#1 or sh-circ#2 transfection, EdU positive cells were reduced, and the trend was rescued by circGLIS3 overexpression (Fig. 1C). Colony formation assay indicated that transfection of sh-circ#1/2 evidently inhibited the proliferation ability of cancer cells, and the inhibitory influence was countered by pcDNA3.1-circ co-transfection (Fig. 1D). Subsequently, we determined the apoptotic capability of cells via terminal deoxynucleotidyl transferase (TdT) dUTP nick-end labeling (TUNEL) assay. Based on the experimental results, we found that inhibition of circGLIS3 facilitated cell apoptosis, and pcDNA3.1-circ co-transfection countervailed the promoting effect (Fig. 1E). All results suggested that knockdown of circGLIS3 could weaken cell proliferation while strengthening cell apoptosis in GBM.

### CircGLIS3 regulates CAPG and GLIS3 in GBM cells

**Fig. 2:**
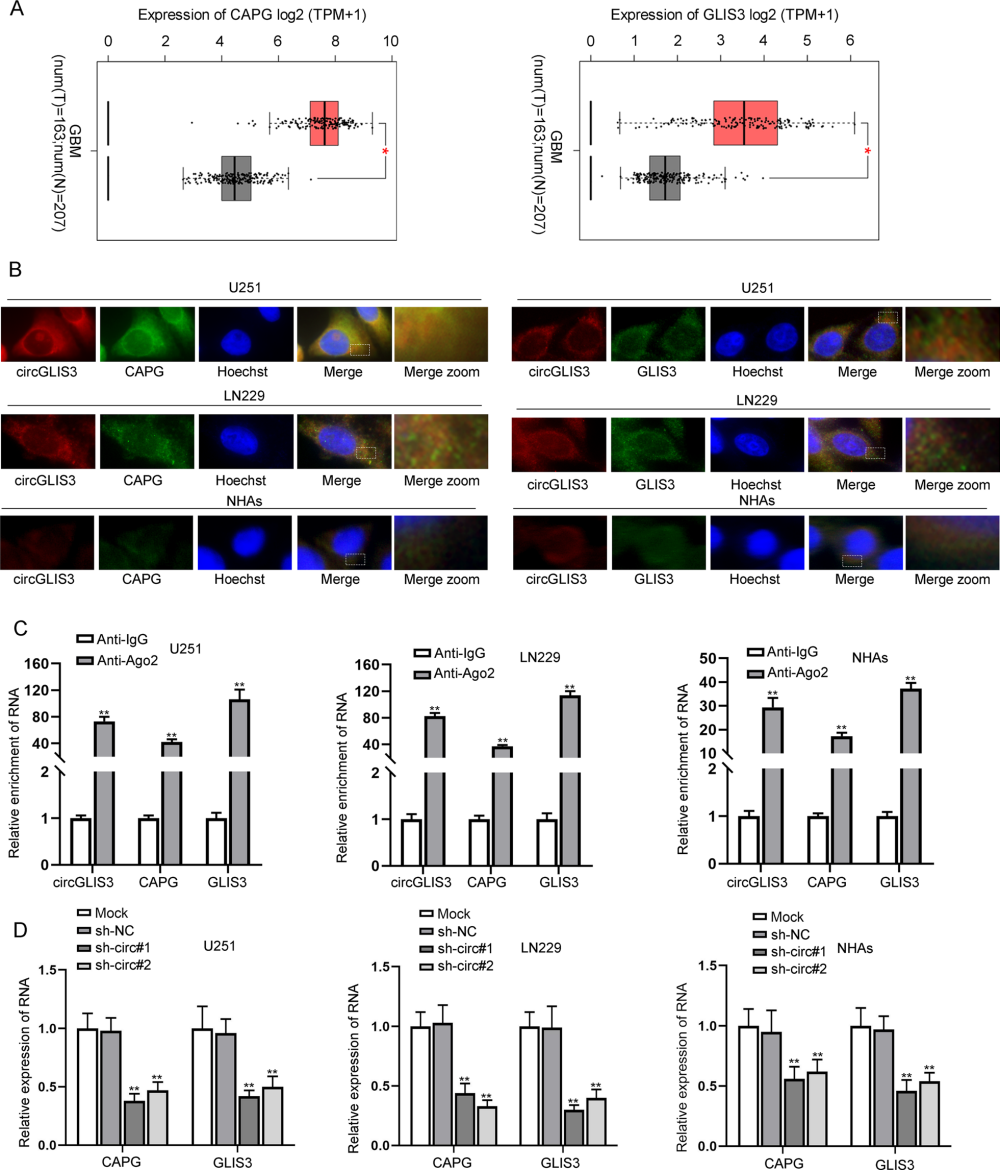
CircGLIS3 regulates CAPG and GLIS3 in GBM cells. **A** The expression levels of CAPG and GLIS3 in GBM tissue and normal deceased tissues were predicted from GEPIA database. **B** FISH was used to detect the co-localization of circGLIS3 and CAPG or GLIS3. **C** RIP assay accompanied with qRT-PCR analysis was carried out to investigate whether circGLIS3, CAPG, and GLIS3 were precipitated in the RISC. **D** QRT-PCR was performed to detect expression of CAPG and GLIS3 in GBM cells and NHAs with circGLIS3 knockdown. Student’s t-test was applied for statistical analysis in **A**/**C** while one-way ANOVA was applied for statistical analysis in **D**. *P < 0.05, **P < 0.01

In this part, we mainly investigated the regulatory mechanism of circGLIS3 in GBM cells. CircRNAs have been reported to regulate downstream mRNAs including their host genes to influence cell phenotype in cancers including gastric carcinoma, clear cell renal cell carcinoma and GBM [28–30]. Therefore, the potential correlation of circGLIS3 with CAPG, a promising biomarker of GBM [31], or with its host gene GLIS3 was then taken into consideration. According to Gene Expression Profiling Interactive Analysis (GEPIA) (http://gepia.cancer-pku.cn/) with data from The Cancer Genome Atlas (TCGA) and Genotype-Tissue Expression (GTEx), CAPG and GLIS3 were both up-regulated in GBM tissues (Fig. 2A). As depicted in Additional file 1: Fig. S2B, both CAPG and GLIS3 were up-regulated in GBM cells. Subsequently, we continued to explore the relationship between circGLIS3 and CAPG and between circGLIS3 and GLIS3. According to fluorescence in situ hybridization (FISH) assay, co-localization of circGLIS3 and CAPG or GLIS3 in cytoplasm was confirmed (Fig. 2B). Previous studies have suggested that cytoplasmic circRNAs could function as a competing endogenous RNA (ceRNA) to sponge miRNAs and regulate mRNA expression (32, 33). Therefore, circGLIS3 was hypothesized to regulate CAPG and GLIS3 via sponging certain miRNA. RNA-induced silencing complexes (RISCs) are formed by miRNA ribonucleoprotein complexes, which is present in anti-Ago2 immunoprecipitates, and anti-Ago2 immunoprecipitates contain miRNAs and their interacting RNA components [34]. On this basis, anti-Ago2 was used for conducting RNA binding protein immunoprecipitation (RIP) assay, and it was observed that circGLIS3, CAPG, and GLIS3 were all precipitated in RISCs, implying the potential involvement of a ceRNA network (Fig. 2C). Then, we found that when circGLIS3 in cells were knocked down, CAPG and GLIS3 mRNA levels were both reduced (Fig. 2D). For further confirmation, we examined the protein levels of CAPG and GLIS3 in U251 and LN229 cells with circGLIS3 knockdown or overexpression, respectively. A decrease in CAPG and GLIS3 protein levels was observed as a result of circGLIS3 silence, while circGLIS3 overexpression led to the opposite consequences (Additional file 1: Fig. S2C). In sum, circGLIS3 could positively regulate CAPG and GLIS3 in GBM cells.

### CircGLIS3 modulates CAPG and GLIS3 in GBM cells by sponging miR-449c-5p

**Fig. 3:**
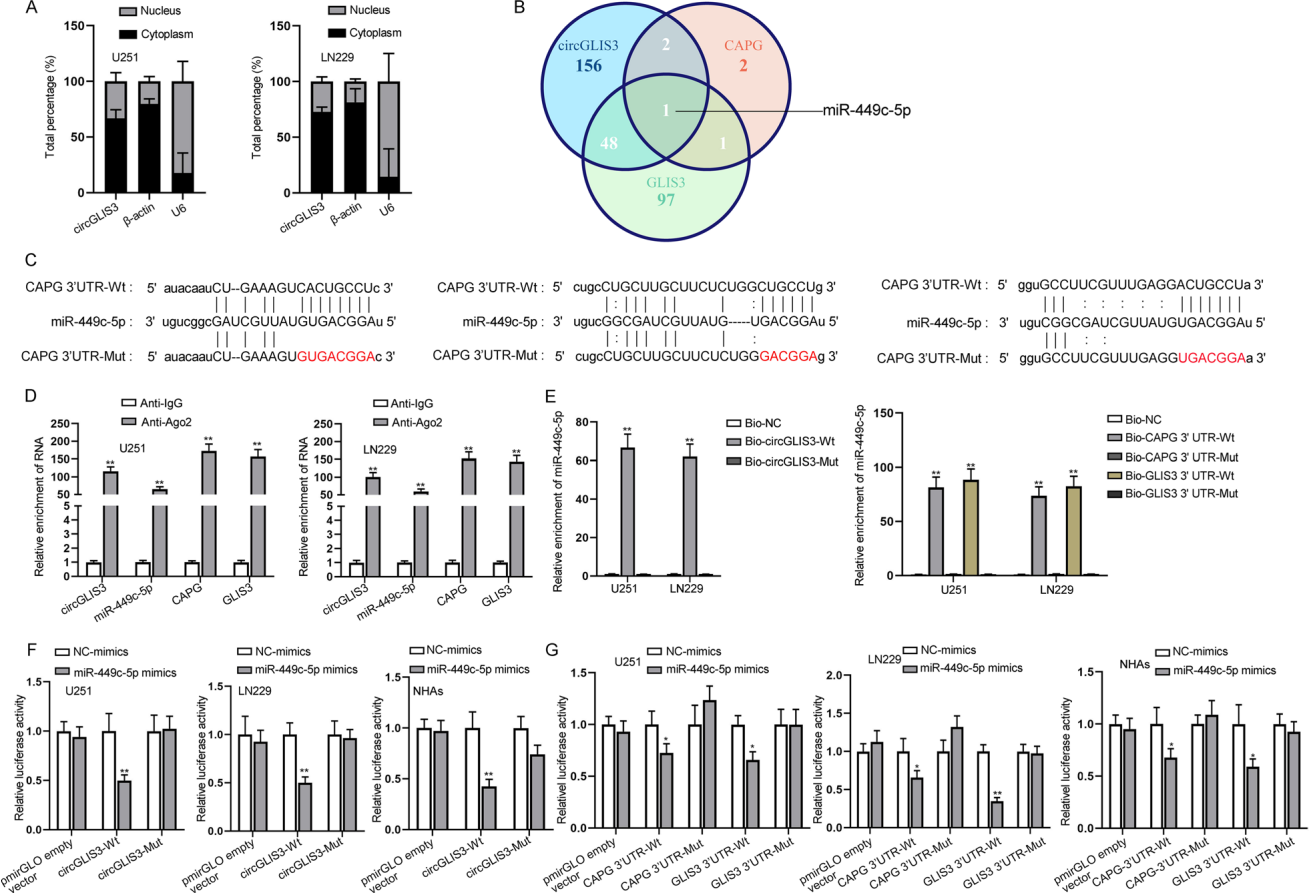
CircGLIS3 regulates CAPG and GLIS3 in GBM cells by sponging miR-449c-5p. **A** Nuclear-cytoplasmic fractionation validated the subcellular position of circGLIS3. **B** starBase database predicted the miRNA binding with circGLIS3, CAPG, and GLIS3. **C** The binding sites between miR-449c-5p and circGLIS3, CAPG 3ʹUTR or GLIS3 3ʹUTR predicted on starBase and the mutant binding sites of circGLIS3, CAPG 3ʹUTR, and GLIS3 3ʹUTR were demonstrated. **D** RIP assay assessed circGLIS3, miR-449c-5p, CAPG, and GLIS3 in cell lysates precipitated with antibodies against IgG and Ago2. **E** Lysates from U251 and LN229 cells were respectively subjected to co-culturing of biotinylated circGLIS3, biotinylated CAPG 3ʹUTR, or biotinylated GLIS3 3ʹUTR in pull down assay. **F**, **G** The luciferase activity in U251 and LN229 cells co-transfected with indicated plasmids was measured to validate the binding relation between miR-449c-5p and circGLIS3/CAPG 3ʹUTR/GLIS3 3ʹUTR at the predicted sites. Student’s t-test was applied for statistical analysis in **D**. One-way ANOVA and two-way ANOVA were respectively applied for statistical analysis in **E** and in **F**, **G**. *P < 0.05, **P < 0.01

The specific ceRNA network of circGLIS3 was next explored. For further confirmation of the subcellular distribution of circGLIS3, subcellular fractionation experiment was first conducted and the result confirmed the major existence of circGLIS3 in cytoplasm (Fig. 3A). After searching on starBase (http://starbase.sysu.edu.cn/), we discovered that miR-449c-5p was the only miRNA potentially binding to circGLIS3, CAPG and GLIS3 (Fig. 3B). Binding sites between miR-449c-5p and circGLIS3, between miR-449c-5p and CAPG 3ʹUTR, and between miR-449c-5p and GLIS3 3ʹUTR were shown in Fig. 3C, and the mutant sequences were also illustrated in red. The result of RIP assay demonstrated that circGLIS3, miR-449c-5p, CAPG and GLIS3 were simultaneously precipitated in anti-Ago2, reflecting their co-existence in RISCs (Fig. 3D). In RNA pull down assay, miR-449c-5p was largely pulled down by biotinylated wild-type circGLIS3, biotinylated wild-type CAPG 3ʹUTR and biotinylated wild-type GLIS3 3ʹUTR, but not by their mutated forms (Fig. 3E). Luciferase reporter assay was then conducted with GBM cell lines (U251 and LN229) and NHAs for further verification of the indicated binding at the predicted sites. Co-transfection of miR-449c-5p mimics weakened the luciferase activity in cells transfected with circGLIS3-Wt, CAPG 3ʹUTR-Wt or GLIS3 3ʹUTR-Wt constructs, while that of luciferase reporters carrying mutant sequences was hardly affected (Fig. 3F, G). Subsequently, we examined the influence of circGLIS3/miR-449c-5p on CAPG and GLIS3 expression via western blot assay. From the result, we could certify that CAPG and GLIS3 protein levels decreased in response to circGLIS3 depletion could be rescued by miR-449c-5p inhibition (Additional file 1: Fig. S2D). Then, functional experiments were carried out in a rescue way to test the influence of circGLIS3/miR-449c-5p on GBM cell proliferation and apoptosis. CCK-8, EdU and colony formation assay jointly reflected that miR-449c-5p overexpression could countervail the promoting effect of circGLIS3 upregulation on GBM cell proliferation (Additional file 1: Fig. S3A–C). TUNEL assay also evidenced that GBM cell apoptosis inhibited by circGLIS3 overexpression was offset by miR-449c-5p augment (Additional file 1: Fig. S3D). In conclusion, circGLIS3 sponged miR-449c-5p to regulate CAPG and GLIS3 expression and affect GBM cell proliferation and apoptosis.

### CircGLIS3/miR-449c-5p/CAPG/GLIS3 axis regulates proliferation and apoptosis of GBM cells

**Fig. 4:**
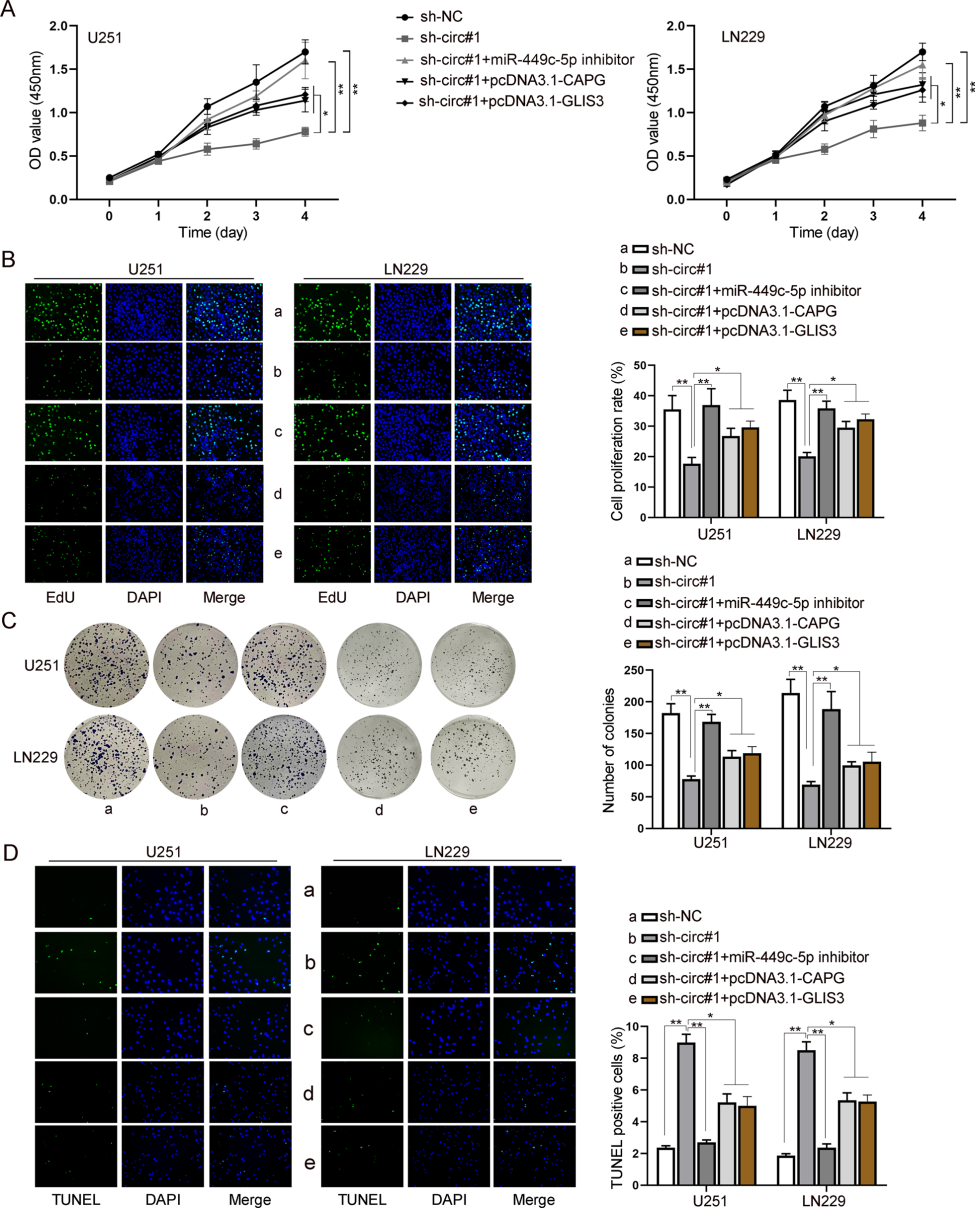
CircGLIS3/miR-449c-5p/CAPG/GLIS3 axis suppresses proliferation of GBM cells. **A** CCK-8 assay was carried out to detect cell viability of U251 and LN229 cells in different groups. **B** EdU assay was used to measure proliferation of U251 and LN229 cells under different conditions. **C** Colony formation assay was employed to detect proliferation of U251 and LN229 cells after transfection of different plasmids. **D** TUNEL assay was used to evaluate apoptosis of U251 and LN229 cells in different groups. One-way ANOVA was applied for statistical analysis. *P < 0.05, **P < 0.01

To further confirm the effect of circGLIS3/miR-449c-5p/CAPG/GLIS3 axis on GBM cells, we conducted functional experiments in a rescue way. Specifically, miR-449c-5p inhibitor (for miR-449c-5p inhibition), pcDNA3.1-CAPG (for CAPG overexpression) or pcDNA3.1-GLIS3 (for GLIS3 overexpression) was designed for the rescue experiments. CCK-8, EdU and colony formation assays were performed to analyze proliferation ability of U251 and LN229 cells after indicated transfection. As shown in Fig. 4A–C, miR-449c-5p inhibition or overexpression of CAPG or GLIS3 reversed the attenuated proliferation ability of U251 and LN229 cells induced by circGLIS3 depletion. In addition, the promoting effect of circGLIS3 knockdown on GBM cell apoptosis could be counteracted by co-transfection of miR-449c-5p inhibitor, pcDNA3.1-CAPG or pcDNA3.1-GLIS3 (Fig. 4D). In summary, we confirmed that circGLIS3 affected the proliferation and apoptosis of GBM cells via miR-449c-5p/CAPG/GLIS3.

## Discussion

Previous studies have demonstrated that in human cancer pathogenesis, circRNAs play important roles [35]. In GBM, the oncogenic or anti-oncogenic role of circRNAs in cancer progression has been found [36].

In this study, through the software prediction, many circRNAs were determined to be dysregulated [37]. Among these circRNAs, circGLIS3 was identified to be remarkably up-regulated in GBM tissues compared with non-tumor controls [37]. A total of 16 isoforms of circGLIS3 were confirmed on circBase. Circ_0005890 was the most up-regulated isoform of the 16 isoforms in GBM cell lines (U251 and LN229) in comparison with NHAs via qRT-PCR analysis (Additional file 1: Fig. S1). A series of functional assays demonstrated that knockdown of circGLIS3 attenuated the viability of GBM cell lines (Fig. 1). Qi et al. discovered that CAPG was the candidate biomarker of GBM [38]. CAPG was identified to be up-regulated in glioblastoma cell lines in previous study [26] and GLIS3 was found to be an overexpressed gene in GBM tissues [39]. GEPIA data also indicated that CAPG and GLIS3 were up-regulated in GBM tissues relative to deceased normal tissues (Fig. 2A). Additionally, the up-regulation of CAPG and GLIS3 in GBM cells relative to NHAs was validated via qRT-PCR analysis (Additional file 1: Fig. S2B). Considering that circGLIS3, CAPG, and GLIS3 were overexpressed in GBM tissues, we found that according to the results from FISH, RIP assay, and qRT-PCR, CAPG and GLIS3 were positively regulated by circGLIS3 and the three genes were co-localized in cell cytoplasm, implying the possibility of a ceRNA network (Fig. 2B–D). MiR-449c-5p served as a suppressor in many types of cancers, such as liver cancer [40], and gastric carcinoma [41]. However, whether miR-449c-5p played a role in GBM was obscure. After further confirming the major accumulation of circGLIS3 in cell cytoplasm (Fig. 3A), we searched on starBase and discovered that only miR-449c-5p was potential to bind with circGLIS3, CAPG, and GLIS3 (Fig. 3B). The binding correlation between miR-449c-5p and the three genes at the predicted sites was respectively validated via RIP assay, RNA pull down assay, and luciferase reporter assay (Fig. 3C–G).

Previous studies have unveiled the important role of circRNAs in human cancers, including GBM [42]. For example, GBM cell proliferation was promoted by circPTN to sponge miR-145-5p/miR-330-5p [43], and additionally, GBM angiogenesis was regulated by FUS/circ_002136/miR-138-5p/SOX13 signaling pathway [44]. It was suggested that circRNA targeted miRNA by acting as a miRNA sponge [45]. In view of the seriousness of GBM to threaten patients’ lives [46], we investigated into regulation of circGLIS3 in proliferation and apoptosis of GBM cells, with CAPG, GLIS3, and miR-449c-5p taken into consideration. In accordance to current study, it was suggested that circGLIS3/miR-449c-5p/CAPG/GLIS3 axis could regulate GBM cell proliferative and apoptotic capacities (Fig. 4).

Taken together, this paper mainly determined circGLIS3 played a role in GBM cell lines via sponging miR-449c-5p to up-regulate CAPG and GLIS3. The regulation of circGLIS3/miR-449c-5p/CAPG/GLIS3 axis was characterized in this research. Accumulating studies have uncovered that circRNAs contributing to stronger proliferation ability and weaker apoptosis ability of cancer cells are also validated to facilitate GBM tumorigenesis and progression in vivo [47, 48]. Moreover, the aberrant expression of genes has been suggested to relate to the prognosis of GBM patients [49, 50]. In this study, animal experiments and clinical samples were lacked, thus the role of circGLIS3 in GBM progression and the association between circGLIS3 expression and the prognosis of GBM patients with could not be investigated. Healthy control tissues were also unavailable at present, which is also a significant limitation of the current study. Therefore, larger-scale clinical research studies are needed to complete those tasks. Additionally, the association among circGLIS3, miR-449c-5p, CAPG, and GLIS3 was validated in both NHAs and GBM cells, indicating that it was not a cancer-specific relationship. Based on previous literature, NHAs could be driven towards a tumor-enhancing phenotype via some molecular networks [51]. Whether the modulatory network involving circGLIS3, miR-449c-5p, CAPG, and GLIS3 could affect the formation of malignant NHAs still needs to be elucidated with further experiments. However, this study still provides new thoughts to the regulatory mechanisms of circRNAs and a novel understanding of GBM pathogenesis.

## Conclusions

The present study showed that circGLIS3 was up-regulated in GBM. The high expression of circGLIS3 led to stronger proliferation ability and weaker apoptosis ability of GBM cells. Knockdown of circGLIS3 suppressed proliferation and advanced apoptosis of GBM cells. CircGLIS3 could exert its impacts on GBM cell proliferative and apoptotic capabilities via miR-449c-5p/CAPG/GLIS3 axis.

## Methods

### Cell lines and cell culture

Human GBM cell lines U251 were acquired from The Cell Bank of Type Culture Collection of Chinese Academy of Sciences. Human GBM cell lines LN229 and normal human astrocytes (NHAs) were obtained from the American Type Culture Collection (Manassas, VA, USA). U251, LN229 and NHAs were incubated in DMEM supplemented with 10% fetal bovine serum and 1% penicillin/streptomycin (Invitrogen). Cells were incubated in a condition with 5% CO_2_ at 37 °C.

### Cell transfection

For inhibition of expression, short hairpin RNAs (shRNAs) targeting circGLIS3 (sh-circ#1/2) and sh-NC (negative control) were constructed by GenePharma (Shanghai, China). Additionally, pcDNA3.1-circGLIS3, pcDNA3.1-CAPG and pcDNA3.1-GLIS3 were obtained for overexpression of circGLIS3, CAPG and GLIS3. NC mimics (the negative control), miR-449c-5p mimics and miR-449c-5p inhibitor (chemically modified, single-stranded RNA molecules designed to eliminate miR-449c-5p activity) were purchased from GenePharma (Shanghai, China). Transfecting the above plasmids into GBM cells (1 × 10^6^) was implemented with the help of Lipofectamine 3000 (Invitrogen). All sequences of indicated transfection plasmids were provided in Additional file 3: Table S1.

### Bioinformatics analysis

circBase software [52] predicted that there were a total of 16 isoforms of circGLIS3. Primer-BLAST [53] was used to design primers of cDNA and gDNA of circGLIS3 (circ_0005890) and cDNA and gDNA of GAPDH. The expression of CAPG and GLIS3 in GBM and normal deceased tissues was obtained from GEPIA [54] based on data from TCGA and GTEx. The binding relation between different factors was predicted on starBase [55].

### Quantitative real-time PCR (qRT-PCR)

By using TRIzol reagent (Invitrogen), total RNA was extracted from cells (1 × 10^5^). Via Bio-Rad CFX96 system (Bio-Rad, CA, USA) with SYBR-Green Real-Time PCR Kit (Toyobo, Osaka, Japan), qRT-PCR was performed. The utilization of Reverse Transcription Kit (Takara, Otsu, Japan) helped RNA to be reversely transcribed into cDNA. 2^−ΔΔCT^ method was used to obtain the relative expressions. β-actin and U6 were used as internal references. All primer sequences and probe sequences, as well as NCBI Accession number of circGLIS3 isoforms, were provided in Additional file 4: Table S2.

### Western blot

Protein lysates were first obtained with RIPA buffer (R0278, Sigma-Aldrich, St. Louis, MO, USA), and PROTTOT-1KT (KGP250, KeyGEN BioTECH, Nanjing, China) was used for extract of total protein, followed by measurement of protein concentration. Later, the proteins treated with SDS-PAGE (1610174, Bio-Rad Laboratories, Shanghai, China) were transferred to PVDF membranes (IPVH00010, Millipore, Bedford, MA, USA), which were blocked with 5% skimmed milk. Primary antibodies including anti-CAPG (HPA019080, Merck, Darmstadt, Germany), anti-GLIS3 (AV39924, Merck) and anti-GAPDH (T6442, Merck) were added for cultivation with membranes overnight at 4 °C and secondary antibodies (07-452, Merck) were then added for further cultivation. Eventually, the proteins were measured via enhanced chemiluminescence (ECL) detection system. Original data were provided in Additional file 2.

### Cell count kit-8 (CCK-8) assay

U251 and LN229 cells were placed onto 96-well plates at a density of 1000 cells per well. Optical density at 450 nm was measured after the addition of CCK-8 reagents (Biolite Biotech, Tianjin, China) at 24, 48, and 72 h.

### 5-Ethynyl-2ʹ-deoxyuridine (EdU) assay

U251 and LN229 cells (1 × 10^5^) were placed onto 96-well plates with RPMI 1640 medium. Then, 100 μl 50 μM EdU reagent was added into the medium for 2-h incubation. Subsequently, paraformaldehyde was adopted to fix the cells and DAPI was used for nuclear staining. The percentage of EdU positive cells was analyzed and quantified through ImageJ.

### Colony formation assay

U251 and LN229 cells were put into plates with 6 wells (1000 cells per well) for incubation. Every 48 h, the medium needed to be changed. After 2-week incubation, the number of cell colonies was calculated manually.

### Terminal deoxynucleotidyl transferase (TdT) dUTP Nick-End labeling (TUNEL) assay

An in-situ cell death detection kit (Roche, Basel, Switzerland) was adopted to gauge cell apoptosis. In brief, 2.5 × 10^5^ cells were blocked with H_2_O_2_ (3% in methanol) for 5 min and then labeled with TdT labeling reaction mix for 1 h at 37 °C. Nuclei exhibiting DNA fragmentation was visualized with DAPI for 15 min and observed under a light microscope (Olympus Corporation, Tokyo, Japan). ImageJ was applied for the measurement of TUNEL positive-stained cells.

### Fluorescence in situ hybridization (FISH)

Hoechst33342 was used to stain nucleus. The RNA FISH probe mix used for circGLIS3, CAPG, and GLIS3 was designed separately. Subsequent to fixation with 4% paraformaldehyde at room temperature, 0.5% TritonX-100 was utilized to permeabilize the cells (5 × 10^4^) for 15 min at 4 °C, followed by PBS washes. Then, cells were co-cultured with the specific probes, and laser scanning confocal microscope (ZEISS) and ImageJ were utilized to analyze samples. Sequences for FISH probes targeting circGLIS3, GLIS3 and CAPG were provided in Additional file 5: Table S3.

### Nuclear-cytoplasmic fractionation

On the premise of manufacturer’s protocol, we used nuclear-cytoplasmic fractionation to determine the subcellular localization of circGLIS3 in cells (1 × 10^7^). PARIS™ kit (Life Technologies, Inc, Gaithersburg, MD, USA) was used to isolate the nuclear and cytoplasmic RNA fractions. β-actin and U6 were employed as internal references for cytoplasm and nucleus respectively. QRT-PCR analysis was conducted to calculate the expression level of circGLIS3 in cytoplasm and nucleus.

### RNA binding protein immunoprecipitation (RIP) assay

RIP assay was conducted with RIP-Assay Kit (MBL Life Science, Shanghai, China). The well-cultured NHAs, U251 and LN229 (6 × 10^7^) were respectively lysed using lysis buffer, and then centrifuged. Cell lysates were incubated with magnetic beads conjugated with anti-Ago2 (Abcam) or the negative control anti-IgG (Abcam) to obtain the protein-RNA complexes. At last, RNAs in the complexes were purified and then quantified via qRT-PCR.

### Luciferase reporter assay

The sequence of CAPG 3ʹUTR, GLIS3 3ʹUTR, or circGLIS3 containing miR-449c-5p binding sites (wild-type) was obtained with the help of bioinformatics tools, and the mutated sequence was chemically synthesized through site directed mutagenesis. Obtained sequences were inserted into pmirGLO luciferase vector (Promega, Madison, WI, USA) respectively. According to assay requirements, NHAs, U251 and LN229 (1 × 10^4^) were transfected with constructed plasmids and NC mimics or miR-449c-5p mimics. Dual Luciferase Reporter Assay System (Promega), with the support of GloMax^®^ 20/20 Luminometer (Promega), was used to measure the luminescence. The relative luciferase activity was normalized to Renilla luciferase activity (internal control). Relevant sequences were provided in Additional file 6: Table S4.

### RNA pull down assay

The purpose of RNA-RNA pull down assay was to detect potential binding relationship between miR-449c-5p and circGLIS3, GLIS3 3ʹUTR or CAPG 3ʹUTR. The biotin-labeled RNA probe targeting circGLIS3 was generated from GenScript Biotech Co., Ltd. (Nanjing, China). Bio-circGLIS3-WT and Bio-circGLIS3-Mut, as well as a biotinylated nonsense sequence as the negative control (Bio-NC), were mixed together with the lysate of U251 and LN229 cells (2 × 10^7^) for 2 h at 4 °C. Then, streptavidin magnetic beads were adopted to incubate with the complexes for 2 h. The level of miR-449c-5p was tested by qRT-PCR. All sequences in this assay were provided in Additional file 7: Table S5.

### Statistical analysis

All representative assays were repeated at least three times. Values were presented as mean ± standard deviation. Statistical analysis was evaluated by the unpaired Student’s t test or analysis of variance (ANOVA) by use of GraphPad Prism 7 (GraphPad Software, Inc., La Jolla, CA). P < 0.05 was thought to have statistical significance.

## Supplementary Information

**Additional file 1: Figure S1. **Characterization of circGLIS3. **Figure S2.** Expression levels of CAPG and GLIS3 in GBM cells. **Figure S3.** Proliferation, migration and apoptosis of GBM cells with circGLIS3 overexpression or co-elevation on circGLIS3 and miR-449c-5p.

**Additional file 2: **Original gel blots for agarose gel electrophoresis (**Figure S1C**) and western blot (**Figure S2C**).

**Additional file 3: Table S1.** Sequences used for gene knockdown and overexpression.

**Additional file 4: Table S2. **Relevant sequences in qRT-PCR.

**Additional file 5: Table S3. **Sequences for FISH probes targeting circGLIS3, CAPG and GLIS3.

**Additional file 6: Table S4.** Sequences of plasmids in luciferase reporter assay.

**Additional file 7: Table S5.** Sequences for synthesizing biotinylated probes used in RNA pull down assay.

## Data Availability

All data generated or analyzed during this study are included in this published article and its additional information files. A total of 16 isoforms of circGLIS3 were obtained from circBase (http://www.circbase.org/). Primer-BLAST (https://www.ncbi.nlm.nih.gov/tools/primer-blast/index.cgi) was used to design primers of cDNA and gDNA of circGLIS3 (circ_0005890) and cDNA and gDNA of GAPDH. The expression of CAPG and GLIS3 in GBM and normal deceased tissues was obtained from GEPIA (http://gepia2.cancer-pku.cn/). The binding relation between different factors was predicted on starBase (https://starbase.sysu.edu.cn/).

## Abbreviations

GBM: Glioblastoma
circRNAs: Circular RNAs
GLIS3: GLIS family zinc finger 3
CAPG: Capping actin protein, gelsolin-like
NHAs: Normal human astrocytes
shRNAs: Short hairpin RNAs
NC: Negative control
qRT-PCR: Quantitative real-time PCR
CCK-8: Cell counting kit-8
EdU: 5-Ethynyl-2ʹ-deoxyuridine
TdT: Terminal deoxynucleotidyl transferase
TUNEL: DUTP Nick-End Labeling
FISH: Fluorescence in situ hybridization
RIP: RNA binding protein immunoprecipitation
ANOVA: Analysis of variance
ceRNA: Competing endogenous RNA
GEPIA: Gene Expression Profiling Interactive Analysis
TCGA: The Cancer Genome Atlas
GTEx: Genotype-Tissue Expression
