# Supplementary material for: A circular RNA derived from GLIS3 accelerates the proliferation of glioblastoma cells through competitively binding with miR-449c-5p to upregulate CAPG and GLIS3
Source: BMC Neurosci. 2022 Sep 16;23:53. doi: 10.1186/s12868-022-00736-6 (PMC9479268; doi:10.1186/s12868-022-00736-6)
Supplement: Supplementary file 1 — Additional file 1: Figure S1. Characterization of circGLIS3. Figure S2. Expression levels of CAPG and GLIS3 in GBM cells. Figure S3. Proliferation, migration and apoptosis of GBM cells with circGLIS3 overexpression or co-elevation on circGLIS3 and miR-449c-5p. [file 12868_2022_736_MOESM1_ESM.docx]

**
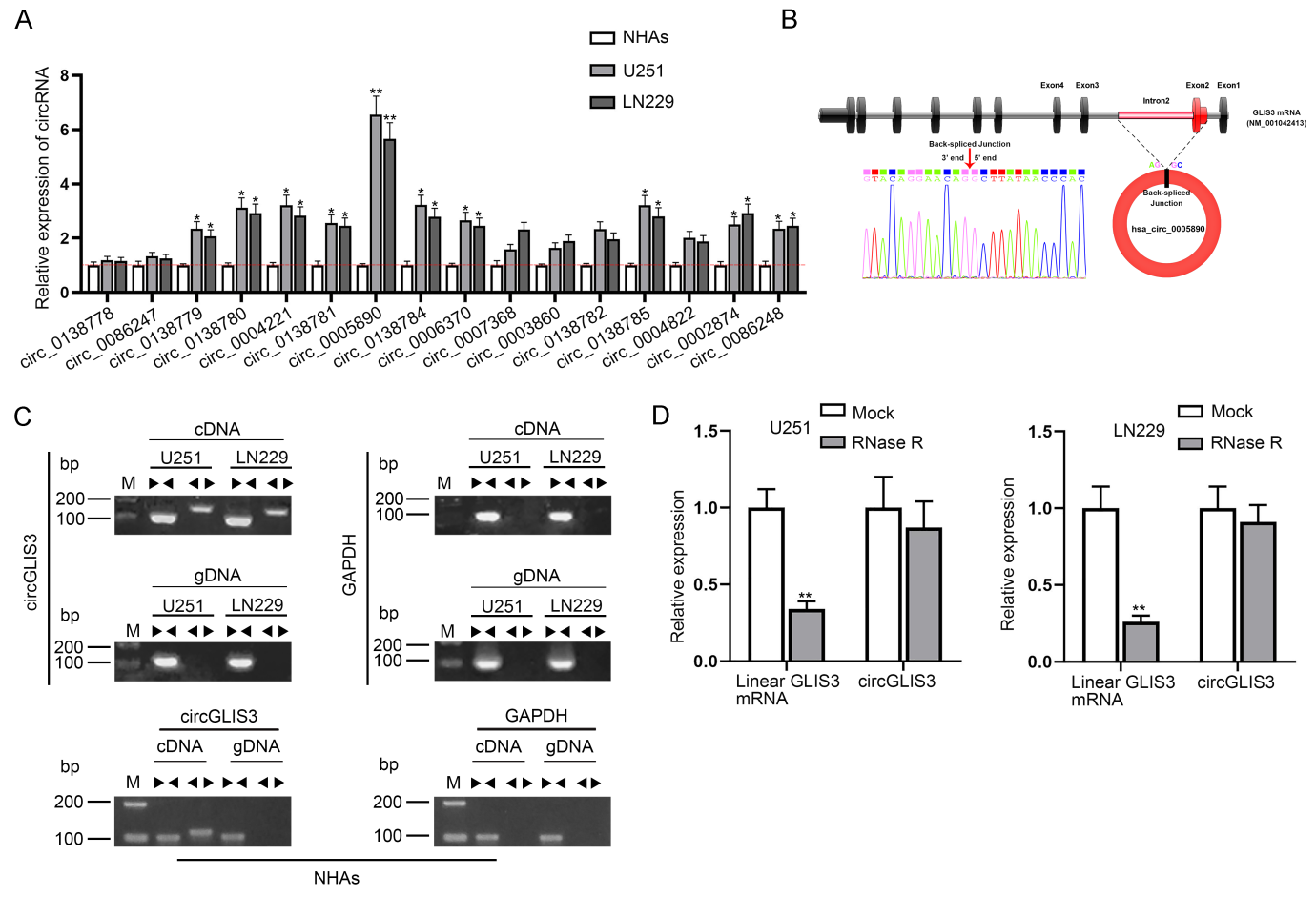
Figure S1** **Characterization of circGLIS3.**

A. Expression levels of 16 circGLIS3 isoforms in NHAs, U251 and LN229 cells were quantified via qRT-PCR. B. Loop formation procedure of circGLIS3 was shown. C. The existence of circGLIS3 was validated by PCR-AGE in NHAs and GBM cell lines. D. Expression of linear GLIS3 mRNA and circGLIS3 in U251 and LN229 cells was measured via qRT-PCR after RNase R treatment. Two-way ANOVA was applied for statistical analysis in Fig. S1A/S1D. *P < 0.05, **P < 0.01.


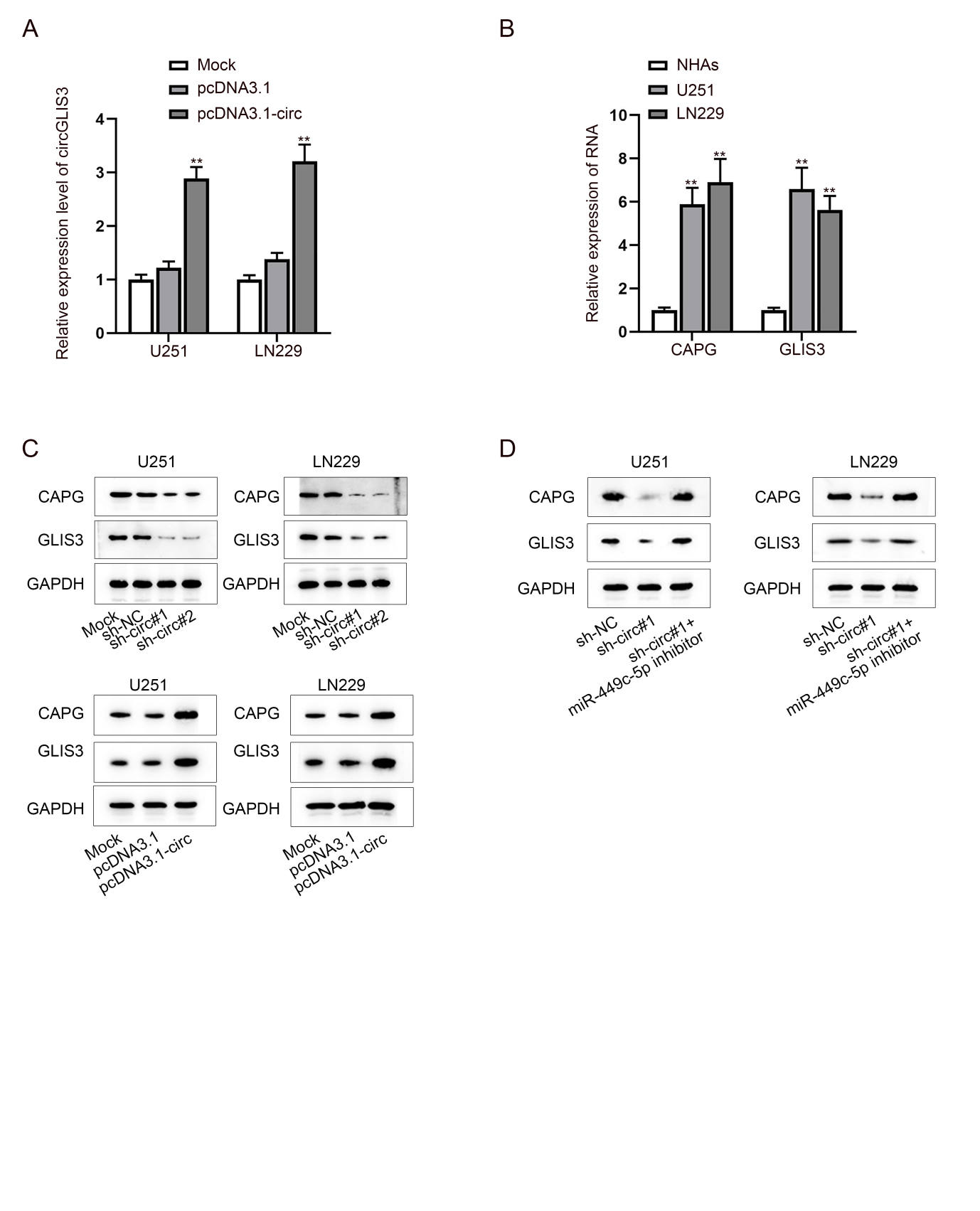
**Figure S2 Expression levels of CAPG and GLIS3 in GBM cells.**

A. The efficiency of pcDNA3.1-circGLIS3 was determined via qRT-PCR. B. Expression of CAPG and GLIS3 in NHAs and GBM cells was examined via qRT-PCR. C. Western blot detected the changes in CAPG and GLIS3 protein levels under circGLIS3 depletion or overexpression. D. CAPG and GLIS3 protein levels upon circGLIS3 knockdown or co-inhibition on circGLIS3 and miR-449c-5p were measured via western blot. One-way ANOVA was utilized to analyze differences among groups in Fig. S2A-B. **P < 0.01.

**
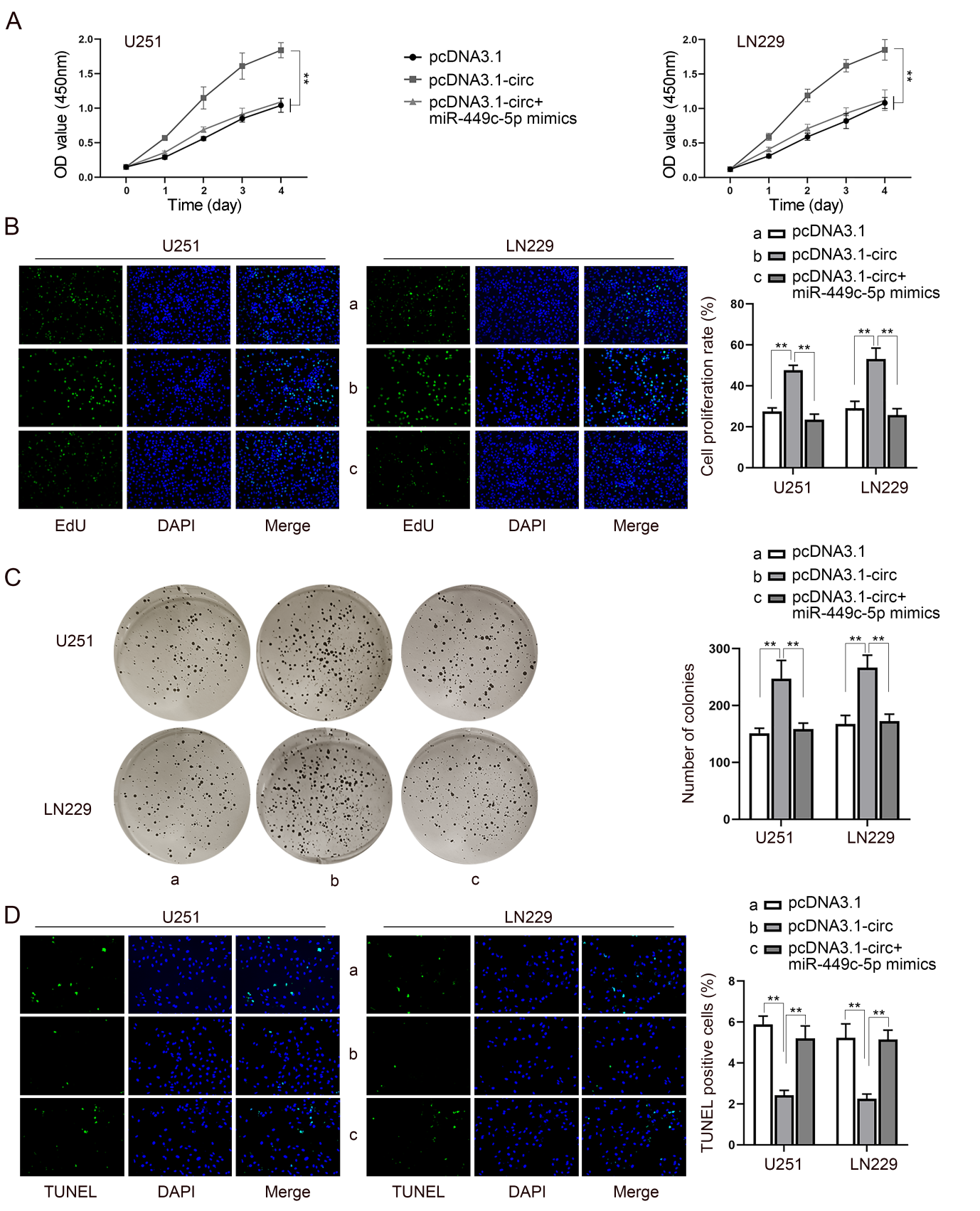
Figure S3 Proliferation, migration and apoptosis of GBM cells with circGLIS3 overexpression or co-elevation on circGLIS3 and miR-449c-5p.**

Rescue experiments were conducted with U251 and LN229 cells respectively transfected with pcDNA3.1, pcDNA3.1-circ or pcDNA3.1-circ+miR-449c-5p mimics. A-C. CCK-8, EdU and colony formation assays were done to assess the alterations in GBM cell proliferation under indicated transfection conditions. D. Cell apoptosis under transfection of indicated plasmids was evaluated via TUNEL. One-way ANOVA was utilized to analyze differences among groups in Fig. S3A-D. **P < 0.01.
